# The impact of family factors and digital technologies on mental health in university students

**DOI:** 10.3389/fpsyg.2024.1433725

**Published:** 2024-08-20

**Authors:** Miguel Ángel Gandarillas, María Natividad Elvira-Zorzo, Gabriela Alicia Pica-Miranda, Bernardita Correa-Concha

**Affiliations:** ^1^Department of Social, Work and Differential Psychology, Faculty of Psychology, Complutense University of Madrid, Pozuelo de Alarcón, Spain; ^2^Department of Social Psychology and Anthropology, Faculty of Psychology, University of Salamanca, Salamanca, Spain; ^3^Vicerrectoría Académica, Dirección General de Asuntos Académicos, Universidad de las Américas, Santiago, Chile

**Keywords:** mental-health, digital-technologies, higher-education, student’s-wellbeing, health-promotion, parenting practices, child rearing

## Abstract

**Introduction:**

A substantial body of research indicates an increasing prevalence of mental health issues among university students in a range of countries. A number of psychosocial factors have been put forward in the research literature as possible explanations for this persistent decline in psychological wellbeing in higher education. The present study focused on the role of family factors and the use of digital technologies by students.

**Methods:**

A replication study was conducted at the University of the Americas (Chile) based on a previous study on psychosocial factors of academic learning patterns and mental health of university students at the Complutense University of Madrid (UCM), Spain. A cross-sectional design was employed, using the same questionnaire, plus indicators of most frequently used digital technologies by the students. The questionnaire was administered online at the same time to all incoming students, gathering a sample of 4,523 students. A series of multiple regressions and ANOVAs was conducted to ascertain the extent to which family and digital factors could be identified as predictors of mental health indicators.

**Results:**

The most significant findings indicate that high levels of parental protection and control/discipline, and especially the high use of social media and smartphones, are particularly salient factors contributing to mental health problems in the learning process of higher education students.

**Discussion:**

The results suggest strategies to promote wellbeing, with a focus on the psychosocial diversity within an inclusive university community. Social and digital innovation, collective entrepreneurship, and participatory place-building may facilitate networks of artistic, cultural, ecological, and sports spaces to promote the sense of university community. A longitudinal follow-up on the same sample across academic years will reveal the extent to which these wellbeing initiatives are fruitful.

## 1 Introduction

The concept of health is not merely the absence of disease; it encompasses a state of holistic well-being across biological, psychological, and social dimensions. This involves heightened self-awareness, functionality, productivity, and community engagement (World Health Organization, 2001, 2014; Vera-Villarroel et al., 2016). In the context of education, it is of paramount importance to examine the mental health status of students, having a significant impact on their learning, academic performance, and overall well-being (Poorolajal et al., 2017; Tran et al., 2017). Among various demographic groups, college students appear to experience a disproportionately high prevalence of mental health issues. Analyses conducted with university students in different countries have revealed a significant proportion of students exhibiting high levels of anxiety, depression, and psychiatric problems (El Ansari et al., 2011; Gallagher, 2014; American College Health Association, 2015; Guassi-Moreira and Telzer, 2015; Lipson et al., 2019). Furthermore, there is a discernible, continuous increase in such conditions over the past 15 years (Lipson et al., 2019; Tan et al., 2023; Haidt, 2024).

Different studies have identified a range of learning-related problems that affect students’ mental wellbeing. These problems include anxiety, irritability, discouragement, apathy, or perceived low self-efficacy, which can affect students’ emotional and psychological well-being and mental health (Matalinares et al., 2016; Aliberti et al., 2019; Batool, 2019; Lew et al., 2019; Morales and Pérez, 2019; Njega et al., 2019; Del Valle et al., 2020; Khalil et al., 2020; MacCann et al., 2020; Tinajero et al., 2020; Trunce et al., 2020; Heritage et al., 2023).

Research on these cognitive and emotional difficulties, as well as their psychosocial stressors, suggests that these difficulties may be exacerbated if not addressed early with inclusive and integrative approaches (e.g., Ibrahim et al., 2013; Santander et al., 2013; Asante and Andoh-Arthur, 2015; Lamis et al., 2016; Samaniego and Buenahora, 2016; January et al., 2018; Lew et al., 2019; Tian-Ci Quek et al., 2019; Trunce et al., 2020; Mirza et al., 2021).

The study of psychosocial predictors of learning-related mental health problems in university students is of great importance for understanding and addressing the challenges they face in their academic and social environment. Identifying these factors provides a sound basis for developing strategies to promote wellbeing and mental health at university level, and to promote the inclusion and achievement of those students with higher mental health problems. This is critical to creating a university environment that supports the academic achievement and emotional well-being of all members, which directly contributes to the overall mental health of the student population. And it is a main goal of the present study, which places a central attention to mental health and wellbeing within the diversity in learning (DinL) in the classroom.

The growing interest in investigating the psychosocial diversity of learning patterns and psychological difficulties within the university setting can be attributed to educators’ desire to achieve a harmonious alignment between their objectives and the increasingly diverse student demographics within class cohorts. This diversity displays a wide range of psycho-educational, social, and contextual traits. A multitude of studies have demonstrated the advantages of addressing diversity in learning styles, habits, strategies, and mental health issues related to learning (referred to here as DinL) from a psychosocial approach. Such initiatives serve as a fundamental tool for promoting collaborative group learning, while simultaneously enhancing social and cultural inclusivity and equality (Manion et al., 2020; Fuentes et al., 2021; Rodríguez-Hidalgo et al., 2021; Rojo-Ramos et al., 2021; Lardy et al., 2022).

A recent study conducted by the authors of this study on the DinL in higher education (Gandarillas et al., 2023) at the Complutense University of Madrid (UCM), Spain, revealed that a significant proportion of students exhibited high levels of mental health problems related to learning, including anxiety (71%), apathy and demotivation (67%), and lack of attention and concentration (55%). The results of the study motivated the research team to explore potential contributing factors that could be associated with the high prevalence of mental health issues observed among university students. To this end, an integrative DinL approach was employed, which considers the psychosocial context of students’ learning patterns and difficulties (Gandarillas et al., 2024). The objective of the present study was to examine the potential influence of psychosocial factors on the observed increase in mental health issues among tertiary education students. In this study, we concentrated on two main areas that have been identified in the research literature as being related to the wellbeing and mental health of young people: family factors and digital information and communication technologies (ICT).

About the influence of family factors on the mental health of students, a substantial body of research exists on the impact of rearing and parenting practices on the development of wellbeing and mental health status in children. Traditionally, research literature classifies child-rearing practices in three main dimensions (Gandarillas, 1995), which are here named Care (affection, warmth, and support in the child’s development), Control (discipline and limits), and Protection. High levels of parental care and involvement appear to be the main positive dimension for developing a good mental health status and academic performance (Fass and Tubman, 2002; Batool, 2019; Dorrance Hall et al., 2020; Kim et al., 2020; Moral et al., 2020). Conversely, high levels of parental control and protection may be related to poor academic adjustment and autonomy (e.g., Robledo and García, 2009; Gordon and Cui, 2012; Ji and Wang, 2018; Njega et al., 2019; Walsh et al., 2023). Overcontrol, as well as lack of control, and excessive overprotection have been linked to an increased risk of depression and anxiety (Maccoby, 1992; Affrunti and Ginsburg, 2012; Franco et al., 2014; Hernesniemi et al., 2017; Gfellner and Córdoba, 2020). In the previous study by the authors of this work at the UCM (Spain), Care appeared negatively related and Control and Protection positively related to mental health problems in learning (Gandarillas et al., 2024).

In terms of parenting styles, the democratic style (which encompasses high levels of support, the encouragement of autonomy and clear boundaries) appears to be the most beneficial for the development of positive psychological patterns in children (Maccoby, 1992; Cortés et al., 2014; Gómez et al., 2014; Fuentes et al., 2015; Gómez et al., 2015; Molina et al., 2017; Jaureguizar et al., 2018; Agbaria and Mahamid, 2023). The remaining parenting styles appear to be more closely associated with an increased vulnerability to anxiety and depression (Franco et al., 2014), which may have an impact on academic performance, up to the university level (Gandarillas et al., 2024).

Another pertinent family factor to consider is the family socio-economic level (Piccolo et al., 2016; Guterman and Neuman, 2018; Martineli et al., 2018; Kim et al., 2020; Rodríguez-Hernández et al., 2020). Low levels of family economic status appear to be generally associated with higher levels of mental health difficulties in academic learning. The relationship may not be linear. In the previous study carried out by the authors of this study in Spain, students from very high family economic status express similar levels of mental health difficulties in their studies as those from families with very low economic status (Gandarillas et al., 2024).

A review of the literature spanning nearly two decades reveals a profound human impact of the technological revolution on the behaviors and well-being of university students. The literature increasingly highlights both positive and negative associations between young people’s use of technology and their mental health and physical well-being (Muñoz-Miralles et al., 2014; García-Oliva et al., 2017; Díaz-Vicario et al., 2019; Emily et al., 2019; Sales et al., 2021; Haidt, 2024). The advent of the COVID−19 pandemic has accelerated the adoption of digital technology by young people, with the online teaching-learning process becoming a prominent feature (Suárez Monzón et al., 2022).

The effects of technologies have been unforeseen and unfold so rapidly that many authors question the individual’s capacity to assimilate them in a healthy way. Indeed, certain digital developments designed to improve the quality of life of the citizen may, in turn, generate discomfort (Forman and Zeebroeck, 2019). A particular example of this is found in the area of high smartphone and social media usage, where the intensity of interactions can contribute to adverse effects on mental health problems, especially in terms of anxiety and self-esteem (Orzech et al., 2016; Rock et al., 2016; Akram and Kumar, 2017; Duke and Montag, 2017; Dhir et al., 2018; Erceg et al., 2018; Hamdi, 2018; Wang and Leif, 2018; Wolfers et al., 2020; Elsayed, 2021; Olorunsogo et al., 2024). Adapting the utilization of digital technology to higher education learning requires a significant investment of time and effort. These elements are fundamental to establishing the foundations for long-term health and well-being (Vaterlaus et al., 2015).

There is a large corpus of research literature underlining the significant influence of parenting practices on the students´ learning process in elementary and secondary education, but there are less studies on to what extent this influence reaches tertiary learning. There are also less studies further analyzing the degree of impact of digital technology affecting mental health issues and wellbeing in higher education students. A combined study of parenting styles and digital technology could yield insights into the differential relevance of both sets of factors in student wellbeing. In order to address these research needs in the research literature, a study was conducted at the University de las Americas (UDLA) in Chile as a replication of the research conducted at the UCM (Spain), testing the relationship between child-rearing practices and digital technologies as possible predictors of mental health indicators in the university learning context. As a primary hypothesis we stated that key family features, such as parenting dimensions and family economic levels, as well as digital tools used by the students may be significantly related to indicators of mental health and psychological difficulties in learning in college students.

## 2 Methods

### 2.1 Design and procedure

A cross-sectional design was employed for the administration of the questionnaire, which was conducted online. The questionnaire was simultaneously administered to all the students admitted to the University that academic year, within a larger admission questionnaire. Students already in the university since previous years were excluded from the study. This was due to the interest in this research project to continue in following years with the same sample within a longitudinal study. Participation in the study was entirely voluntary and confidential, including the informed consent. This work adhered to ethical procedures in accordance with the Declaration of Helsinki (World Medical Association, 2013) and received approval from the Ethical Committee of the UCM (ref n° CE_20211118-15_SOC).

### 2.2 Sample

The participants were 4,523 students, 45% of all the students invited to participate in the study, who represented all academic areas of the university (social sciences, humanities, arts, health and natural sciences, technology, engineering, mathematics) in proportional numbers, with 65% female and 35% male, and a mean age of 25.05 years (*standard deviation* = 8.30).

### 2.3 Instrument

The items on mental health in the study were selected from the authors’ Diversity-in-Learning (DinL) scale. DinL is a self-administered scale that assesses the main dimensions that describe the diversity of learning patterns observed in the classroom, with optimal psychometric properties (Gandarillas et al., 2024). The DinL scale comprises 28 items, each of which is rated on a 4-point Likert scale (1 = Nothing or very little, 2 = Some, 3 = Quite, 4 = A lot). The scale assesses five learning dimensions: *Coping with Difficulties*, *Effort*, *Autonomy*, *Learning by Understanding*, *and Social Influence*. A detailed account of the procedure used to construct the scale is provided in Gandarillas (2022). The items related to mental health are included in the dimension *Coping with Difficulties*, measuring the levels of coping with (most frequent) psychological difficulties in learning. In this study *Coping with Difficulties* showed a Cronbach’s Alpha of 0.81, and a Kaiser-Meyer-Olkin (KMO) value of 0.84. For the purpose of this study four single items were selected from this dimension for further analysis: (1) Levels of anxiety; (2) Bad mood/irritability; (3) Difficulties in attention and concentration; and (4) Apathy/discouragement/lack of motivation, keeping good psychometric properties in the present research. Here, these items showed factor loadings between 0.60 and 0.71 in its dimension (*Coping with Difficulties*). Chi-square tests carried out in each item showed significance levels below 0.001 in all four items. Asymmetry and kurtosis of each item are included in Table 1.

**Table 1:** Correlations between the variables and descriptives (*N* = 4,523).

		1	2	3	4	5	6	7	8	9	10	11	12	13
1. Bad mood/irritability	Person correlation	--												
	Sig. (bilateral)													
2. Levels of anxiety	Person correlation	0.520^**^	--											
	Sig. (bilateral)	0.000												
3. Lack of motivation	Person correlation	0.567^**^	0.451^**^	--										
	Sig. (bilateral)	0.000	<0.001											
4. Difficulties in attention and concentration	Person correlation	0.400^**^	0.453^**^	0.477^**^	--									
	Sig. (bilateral)	<0.001	<0.001	<0.001										
5. Parents´ Support	Person correlation	0.001	–0.016	–0.022	–0.068^**^	--								
	Sig. (bilateral)	0.963	0.272	0.131	<0.001									
6. Parents´ Control	Person correlation	0.112^**^	0.105^**^	0.092^**^	0.102^**^	–0.154^**^	--							
	Sig. (bilateral)	<0.001	<0.001	<0.001	<0.001	<0.001								
7. Parents´ Protection	Person correlation	0.111^**^	0.122^**^	0.092^**^	0.114^**^	0.158^**^	0.248^**^	--						
	Sig. (bilateral)	<0.001	<0.001	<0.001	<0.001	<0.001	<0.001							
8. Gamification apps	Person correlation	0.087^**^	0.091^**^	0.088^**^	0.062^**^	0.161^**^	0.004	0.047^**^	--					
	Sig. (bilateral)	<0.001	<0.001	<0.001	<0.001	<0.001	0.811	0.002						
9. Apps of collaboration with other students	Person correlation	–0.013	–0.001	–0.023	–0.025	0.174^**^	0.018	0.058^**^	0.377^**^	--				
	Sig. (bilateral)	0.364	0.948	0.127	0.092	<0.001	0.214	<0.001	<0.001					
10. Online classes	Person correlation	0.013	0.049^**^	–0.027	–0.020	0.154^**^	0.025	0.041^**^	0.227^**^	0.389^**^	--			
	Sig. (bilateral)	0.395	0.001	0.073	0.178	<0.001	0.097	0.005	<0.001	<0.001				
11. Social media	Person correlation	0.110^**^	0.146^**^	0.065^**^	0.074^**^	0.120^**^	0.031^*^	0.085^**^	0.146^**^	0.189^**^	0.218^**^	--		
	Sig. (bilateral)	<0.001	<0.001	<0.001	<0.001	<0.001	0.039	<0.001	<0.001	<0.001	<0.001			
12. Use of cell phone (hours/day)	Person correlation	0.131^**^	0.130^**^	0.111^**^	0.108^**^	0.005	0.004	0.029	0.021	–0.023	–0.012	0.210^**^	--	
	Sig. (bilateral)	<0.001	<0.001	<0.001	<0.001	0.756	0.795	0.054	0.154	0.114	0.410	<0.001		
13. Age	Person correlation	–0.270^**^	–0.286^**^	–0.269^**^	–0.249^**^	–0.154^**^	0.058^**^	–0.095^**^	–0.264^**^	–0.061^**^	–0.019	–0.165^**^	–0.154^**^	
	Sig. (bilateral)	<0.001	<0.001	<0.001	<0.001	<0.001	<0.001	<0.001	<0.001	<0.001	0.192	<0.001	<0.001	
Descriptives	Mean	2.11	2.80	2.07	2.43	2.40	1.67	2.28	1.63	1.97	2.47	3.17	3.93	25.05
	SD	0.97	0.96	1.03	1.00	0.89	0.81	0.94	0.87	0.96	1.01	0.89	1.16	8.30
	Asymmetry	0.50	–0.28	0.56	0.19	0.16	1.13	0.26	1.25	0.61	0.00	–0.84	0.15	1.48
	Kurtosis	–0.72	–0.92	–0.86	–1.04	–0.92	0.51	–0.91	0.63	–0.71	–1.09	–0.16	–0.46	1.79

The online administered questionnaire included also representative items of the child-rearing dimensions of Care, Control, and Protection. These items were selected from the Egna Minnen av. Barndoms Uppfostran (EMBU) scale, a retrospective Likert-scale questionnaire showing robust internal consistency and factorial validity in diverse contexts and populations (e.g., Arrindell et al., 1988, 2005; Yangzong et al., 2016; Mathieu et al., 2020; Cheng and Wu, 2021; Yongmei and Jiaying, 2022). It measures the three major child-rearing dimensions (named by the authors as Warmth, Rejection and Protection, representing the Care, Control and Protection dimensions, respectively). Two representative items of the Care dimension (the most relevant child-rearing dimension) were selected, as well as one representative item of Control and another item of Protection for mother and father. The items were selected based on conceptual (being most related to the goals of the study) and on statistical criteria. Previous studies using the selected items showed optimal psychometric properties, high statistical representativeness in their respective dimensions and good predictive validity (Gandarillas, 1995, 2011, 2022; Gandarillas et al., 2024). The students were asked to score on the items on rearing practices to them between the ages of 13 and 17 years old, and also on the family’s economic status. Additionally, five items on the frequency of use of frequent digital tools used in their studies were included: Gamification apps (e.g., Kahoot!), apps of online collaboration with other students, online diachronic classes (previously recorded by the professor), social media and networks (e.g., WhatsApp, TikTok, Facebook, Twitter), and use of smartphone. Four socio-demographic variables (biological sex, age, grade, and field of study) were also included (see Appendix for the items used in the study).

### 2.4 Data analysis

Descriptive analyses (mean, standard deviation, asymmetry, and kurtosis) and bivariate Pearson correlations of the items under study were conducted. The means of father’s and mother’s rearing items were used as one variable in the analyses. Also, the mean of the two *Care* items was used as one variable. To estimate the predictive value of the parenting dimensions in the mental health indicators, linear multiple regressions (forward stepwise method) were carried out with parental rearing factors and family economy as predictors (independent variables, IVs) and the selected mental health items as dependent variables (DVs). Another set of linear multiple regressions (forward stepwise method) was carried out with the digital ICT items as predictors with the same DVs as the previous regressions. The *R^2^* determination coefficient, the non-standardized coefficient (*B*), standardized coefficients (*β*), VIF indices and tolerance were also obtained. Assumptions of linearity, normality, homoscedasticity, and multicollinearity were analyzed. To further validate the significant results of the multiple regressions, one-way analyses of variance (ANOVAs) were carried out with the significant predictors of the regressions as IVs (grouped in two levels, low and high, divided by the percentile 50) and the mental health indicators as DVs. In all analyses, results with *p* levels below,05 were considered statistically significant. Data analysis was conducted with the computer programs SPSS (version 27).

## 3 Results

Descriptive statistics and Pearson bivariate correlations between the variables are shown in Table 1. The indices of asymmetry and kurtosis demonstrated values ±1.79, indicating a normal distribution (Mardia, 1970). Correlations did not show evidence of multicollinearity between the key predicting variables. In the multiple regressions on parenting factors, they were found to significantly predict all mental health indicators (see Table 2). In all these multiple regressions, the VIF and the tolerance indices allow the rejection of collinearity of the variables. Of particular interest were the parenting factors of Control and Protection, with positive relationships with expressed mental health problems. The parenting factor of Care did not demonstrate such significant levels, except for difficulties in attention and concentration (negative relationship). Protection emerged as the most significant parenting factor in predicting the mental health indicators. The results of the ANOVAs provided further support for those of the multiple regressions (see Table 3). Family economic levels did not appear to be a significant predictor in any mental health indicator.

**Table 2:** Multiple regressions. Family factors predicting main indicators related to mental health in learning of the university students (*N* = 4523).

Anxiety levels							
Model		*B*	Beta	*t*	Sig.	Tolerance	VIF
2	Constant	2.406		56.56	0.000		
	Parents’ protection	0.104	0.103	6.74	<0.001	0.97	1.03
	Parents’ control	0.094	0.080	5.24	<0.001	0.97	1.03
*R*^2^ = 0.021							

Lack of motivation							
Model		*B*	Beta	*t*	Sig.	Tolerance	VIF
2	Constant	1.728		37.78	<0.001		
	Parents’ protection	0.080	0.074	4.84	<0.001	0.97	1.03
	Parents’ control	0.093	0.074	4.82	<0.001	0.97	1.03
*R*^2^ = 0.021							

Difficulties in attention and concentration							
Model		*B*	Beta	*t*	Sig.	Tolerance	VIF
3	Constant	2.241		37.47	<0.001		
	Parents’ protection	0.117	0.110	7.12	<0.001	0.97	1.04
	Parents’ support	–0.086	–0.076	–5.00	<0.001	0.98	1.02
	Parents’ control	0.078	0.063	4.04	<0.001	0.96	1.04
*R*^2^ = 0,024							

Bad mood/irritability							
Model		*B*	Beta	*t*	Sig.	Tolerance	VIF
2	Constant	1.726		40.15	<0.001		
	Parents’ control	0.107	0.090	5.89	<0.001	0.97	1.03
	Parents’ protection	0.091	0.089	5.87	<0.001	0.97	1.03
*R*^2^ = 0.014							

**Table 3:** Significant (*p* < 0.05) one-factor analyses of variance (ANOVAs) of mental health indicators of the university students by family factors (grouped in low and high) that are significant in the multiple regressions (*N* = 4,522).

Family factors	Mental health indicators	Levels on family factors	Means on mental health	df	*F*	*p*
Parents’ control	Difficulties in attention and concentration	Low	2.37	1/4521	29.72	<0.001
		High	2.53			
	Bad mood/irritability	Low	2.04	1/4521	39.26	<0.001
		High	2.22			
	Anxiety	Low	2.73	1/4521	31.82	<0.001
		High	2.90			
	Lack of motivation	Low	2.01	1/4521	24.48	<0.001
		High	2.16			
Parents’ protection	Difficulties in attention and concentration	Low	2.33	1/4521	50.31	<0.001
		High	2.55			
	Bad mood/irritability	Low	2.03	1/4521	41.59	<0.001
		High	2.21			
	Anxiety	Low	2.71	1/4521	48.08	<0.001
		High	2.91			
	Lack of motivation	Low	2.00	1/4521	25.70	<0.001
		High	2.15			
Parents’ care	Difficulties in attention and concentration	Low	2.48	1/4521	15.28	<0.001
		High	2.36			

df, degrees of freedom.

The multiple regressions on the use of digital technologies also demonstrated significant predictions with all mental health indicators, as evidenced by Table 4. In all the regressions, the VIF and the tolerance indices allow the rejection of collinearity of the variables. Especially relevant was the positive relationships observed between the frequency of social media use and anxiety levels, and bad mood/irritability, both in the multiple regressions and in the ANOVAs (see Table 5). The use of smartphones was also identified as a significant predictor of mental health problems, with gamification apps emerging as a close second. The results indicated that collaborative apps and online diachronic lecturing exhibited some negative predictions with respect to mental health problems. However, these did not yield any significant results in the ANOVAs.

**Table 4:** Multiple regressions. Digital technologies predicting main indicators of mental health in learning (*N* = 4,523).

Anxiety levels Model		*B*	Beta	*t*	*p*	Tolerance	VIF
4	Constant	2.005		29.246	<0.001		
	Social media	0.131	0.122	8.018	<0.001	0.91	1.09
	Smartphone use	0.083	0.101	6.730	<0.001	0.95	1.05
	Gamification apps	0.101	0.092	5.846	<0.001	0.85	1.17
	Apps for online collaboration with other students	−0.056	−0.057	−3.550	<0.001	0.83	1.19
	*R*^2^ = 0.039						

Lack of motivation Model		*B*	Beta	*t*	*p*	Tolerance	VIF
5	Constant	1.564		20.406	<0.001		
	Smartphone use	0.086	0.097	6.439	<0.001	0.95	1.05
	Gamification apps	0.127	0.109	6.806	<0.001	0.85	1.18
	Online collaboration apps with other students	−0.059	−0.055	−3.260	0.001	0.75	1.32
	Social media	0.054	0.047	3.044	0.002	0.89	1.12
	Online diachronic classes	−0.040	−0.039	−2.406	0.016	0.82	1.22
	*R*^2^ = 0.026						

Difficulties in attention and concentration Model		*B*	Beta	*t*	*p*	Tolerance	VIF
4	Constant	1.905		26.231	<0.001		
	Smartphone use	0.080	0.093	6.169	<0.001	0.95	1.05
	Gamification apps	0.086	0.075	4.714	<0.001	0.85	1.17
	Online collaboration apps with other students	−0.065	−0.062	−3.832	<0.001	0.84	1.19
	Social media	0.062	0.055	3.597	<0.001	0.91	1.09
	*R*^2^ = 0.020						

Bad mood/Irritability Model		*B*	Beta	*t*	*p*	Tolerance	VIF
4	Constant	1.418		19.701	<0.001		
	Gamification apps	0.106	0.096	6.047	<0.001	0.85	1.17
	Apps of collaboration with other students	–0.063	−0.062	−3.705	<0.001	0.84	1.19
	Social media	0.092	0.085	5.476	<0.001	0.91	1.09
	Use of cell phone (hours / day)	0.091	0.110	7.305	<0.001	0.95	1.05
*R*^2^ = 0.033							

**Table 5:** Significant (*p* < 0.05) one-factor analyses of variance (ANOVAs) of mental health indicators of the university students by digital technologies (grouped in low and high) that are significant in the multiple regressions (*N* = 4,522).

Digital technologies	Mental health indicators	Levels on family factors	Means on mental health	*df*	*F*	*p*
Social media	Difficulties in attention and concentration	Low	2.36	1/4521	31.76	<0.001
		High	2.53			
	Bad mood/irritability	Low	2.01	1/4521	70.77	<0.001
		High	2.25			
	Anxiety	Low	2.67	1/4521	107.15	<0.001
		High	2.96			
	Lack of motivation	Low	2.00	1/4521	23.08	<0.001
		High	2.15			
Use of smart phone (hours per day)	Difficulties in attention and concentration	Low	2.39	1/4521	22.18	<0.001
		High	2.55			
	Bad mood/irritability	Low	2.07	1/4521	25.84	<0.001
		High	2.23			
	Anxiety	Low	2.76	1/4521	22.86	<0.001
		High	2.91			
	Lack of motivation	Low	2.03	1/4521	19.07	<0.001
		High	2.17			
Gamification apps	Difficulties in attention and concentration	Low	2.38	1/4521	18.21	<0.001
		High	2.51			
	Bad mood / irritability	Low	2.05	1/4521	30.23	<0.001
		High	2.21			
	Anxiety	Low	2.73	1/4521	35.55	<0.001
		High	2.90			
	Lack of motivation	Low	1.97	1/4521	54.03	<0.001
		High	2.20			

df, degrees of freedom.

Anxiety levels were identified as the most significant mental health problem, exhibiting the highest mean (Table 1). This was predicted to be highest, particularly in relation to digital technologies, and showed the highest differences (especially between low and high frequency of use of social media) in the ANOVAs. Bad mood/irritability was identified as the second most significant indicator, particularly in relation to digital technologies.

In general terms, digital technologies showed higher predictions and differences than family factors in mental health indicators.

## 4 Discussion

The results generally supported the main hypothesis. The multiple regressions indicated that both parenting patterns and digital technologies were significant predictors of mental health indicators in learning. While the R^2^ values were not especially high, the predictors were found to be highly significant. The significant ANOVAs provided additional validity to the results.

The parental factors and digital technologies showed significant predictions and differences in the levels of mental health indicators of those included in the study. In regards to the parental factors, results on the positive relationship between Control and Protection and indicators of mental health problems supported the results obtained in the sample at the UCM (Spain), with parental Protection as the most relevant predictor of mental health status (Gandarillas et al., 2024). The literature indicates that an increase in parental over-protection over the past few decades may be a contributing factor to the decrease on coping skills in the face of frustration, stress, and anxiety (Affrunti and Ginsburg, 2012; Hernesniemi et al., 2017; Gfellner and Córdoba, 2020). Furthermore, higher levels of parental Control have been linked to higher rates of mental health problems in learning, as evidenced by numerous studies in the field (Robledo and García, 2009; Gordon and Cui, 2012; Ji and Wang, 2018; Njega et al., 2019).

The results of this study support the authoritative parenting style as a more positive approach to fostering mental health in students, in line with previous findings in this field (Franco et al., 2014). However, the lack of statistical significance of parental Care as a predictor of student mental health indicators is not consistent with most of the research literature (e.g., Fass and Tubman, 2002; Batool, 2019; Dorrance Hall et al., 2020; Kim et al., 2020; Moral et al., 2020; Walsh et al., 2023) and with our previous study in Spain. Similarly, family economy did not demonstrate any significant prediction on mental health indicators, a result that does not align with the general findings in the literature (Piccolo et al., 2016; Guterman and Neuman, 2018; Martineli et al., 2018; Kim et al., 2020; Rodríguez-Hernández et al., 2020). One potential explanation for this result might be related to the high social-economic homogeneity of the sample in the UDLA, a private university. The previous study in the UCM (a public university with higher social-economic diversity) found that students in families with the lowest and highest economic levels expressed higher mental health problems in learning, which may be related to parenting patterns (Gandarillas et al., 2024).

The utilization of digital technologies by the students exhibited a robust relationship with the prevalence of mental health issues among university students. In particular, the frequency of social media usage demonstrated a highly significant association with all mental health indicators included in the study, with the highest relationship observed in anxiety levels. Social media use emerged as the single most influential variable in the relationship with anxiety in this study, surpassing other digital technologies and family factors. The negative impact of social media on mental health has been documented in numerous studies over recent years (e.g., Álvarez and Moral, 2020; Regalado Chamorro et al., 2022). This study also identified the high use of smartphones as an important factor influencing the impact on mental health, in line with the findings of recent research (e.g., Wang and Leif, 2018; Olorunsogo et al., 2024). Furthermore, social media appears to be particularly influential in the context of negative affect, including feelings of bad mood and irritability. Social media may contribute to the development of negative affects through two main mechanisms. Firstly, it may facilitate a tendency to engage in comparisons with others that are perceived as more successful or attractive, which can lead to feelings of frustration and inadequacy. Secondly, social media may facilitate the formation of negative attitudes toward outgroups, which can result in discriminatory biases, negative feelings and hatred (e.g., Álvarez and Moral, 2020; Moreira de Freitas et al., 2021; Mi et al., 2023). In recent years, the potential psychosocial mechanisms underlying the effects of digital technology on mental health have been the subject of extensive research. Several factors that may contribute to anxiety and hatred, including the phenomenon of nomophobia (Wolfers et al., 2020), fear of missing out (FOMO) (Hodkinson, 2019), and digital hatred (Walther, 2022), have been identified. The use of social media may serve to amplify ingroup biases, with phenomena such as the ‘echo chamber,’ ‘filter bubbles,’ and the ‘confirmation bias’ being related to well-known processes of groupthink, conformity, or polarization, motivated by the need of the digital group members to be accepted or to get higher status in the group or by the fear to be excluded (Walther, 2022). These biases may then lead to emotional and affective states that feedback such biases (Gandarillas and Montañes, 2019; Törnberg et al., 2021; Heylighen and Beigi, 2023).

Anxiety is the most prevalent mental health issue among university students, according to the findings of this research. It is more strongly associated with digital technologies than with family factors. Our study revealed a significant relationship between the frequency of social media use and anxiety, more pronounced than with any family factor. Just a few years ago, it would have been challenging to imagine that a digital platform for sharing texts, images, and videos could have a more profound impact on the mental health of young people than parental rearing patterns. The findings of various studies indicating a significant increase in anxiety levels among youth over the past 15 years are a cause for concern in different countries (Wolfers et al., 2020; Tan et al., 2023; Haidt, 2024). This study suggests that the high use of certain digital platforms and devices may contribute more to this rise in anxiety levels than other factors such as parenting patterns.

An alternative explanation for the results on the relationship between significant digital technologies and mental health problems may not imply a cause-and-effect relationship. For example, it is possible to hypothesized that individuals with trait anxiety may be more prone to the abuse of social media and smartphones. However, this interpretation is difficult to sustain as we would also need to explain how individuals with high levels of irritability, low levels of motivation, and difficulties in attention and concentration are also more prone to high levels of social media and smartphone use. Furthermore, the significant negative correlations between age and mental health indicators and digital technologies provide additional support for the causal influence of digital technologies (especially social media and smartphone use) on mental health status. The results suggest that younger students tend to utilize digital devices and platforms to a greater extent and exhibit higher levels of mental health issues in the context of learning.

The elements included in this study explained only a portion of the variance in mental health indicators. Furthermore, other psychosocial factors may also affect mental health and wellbeing in the learning process, such as different academic demands and methods, the recent impact of the COVID-19 pandemic, or peer competition (e.g., El Madani et al., 2023; Nagib et al., 2023). Nevertheless, the general results of this study are unambiguous and permit the drawing of firm conclusions. These results indicate the necessity for the development of tools and strategies to foster individual and collective wellbeing. These issues not only affect students, but also the social environment in which they operate. Therefore, it is of the utmost importance to consider the dynamics of the university and socio-cultural environment in order to effectively address problems such as depression, anxiety and substance abuse, among others (Nahar et al., 2023).

At the universities of the research teams participating in this study, there has been a growing concern about the mental health of students. This has led to the implementation of a series of strategies and proposals designed to promote wellbeing and prevent mental health problems. These strategies are outlined next.

The University of the Americas in Chile (UDLA) offers comprehensive academic support strategies for new students through the Integrated Student Support System (ISSS). This system is designed to ensure the wellbeing and academic achievement of students from admission to graduation. It is adaptable to the needs of new students in a first stage, providing a comprehensive framework that covers academic, financial, and psychosocial aspects. A key component of the ISSS is the Diagnostic Assessment, which includes disciplinary and psychoeducational assessments in areas such as communication skills, mathematical, scientific, and technological thinking, as well as learning strategies. This evaluation is applied to all students at the beginning of their university life, allowing needs to be identified. Personalized support plans are developed that include peer tutoring, mentoring, remedial courses, and specific workshops to strengthen the areas where the student needs more support.

UDLA’s dedication to student retention is evidenced by the Dropout Risk Alert System (DRAS), a component of the ISSS that monitors student progress throughout the semester. DRAS employs a multivariate-based predictive model to identify students with psychological difficulties and at risk of dropping out and deploys early interventions to address identified issues. This ensures that students receive the support necessary to continue and complete their education. The UDLA also endeavors to ensure the well-being of its students through the Student Wellbeing Program, which is part of the ISSS department. This program offers a range of services, including health services, psychological counseling, sports, and cultural activities, with the aim of fostering the comprehensive development of students. This integrated support system reflects the proactive and student-centered approach, a fundamental principle of the UDLA educational model, ensuring that all students, especially new students, have the necessary tools and resources to be successful in their academic and professional careers.

To provide support to students with different psychological problems, the Universidad Complutense de Madrid (UCM) offers a University Clinic, which provides psychotherapy and workshops to promote wellbeing and to prevent psychopathologies. The UCM also offers the PsiCall program, a telephone service for psychological support. The University runs annual initiatives to promote wellbeing and mental health within the university community, leading to a variety of small innovative projects. Moreover, a large mental health screening study is carried out, reaching all university students and including a broad range of mental health indicators and psychosocial correlating factors in an online questionnaire. Based on the results of the students’ responses to the questionnaire, the digital application provides feedback, recommending different preventive activities or clinical treatments within the university.

**Figure 1:**
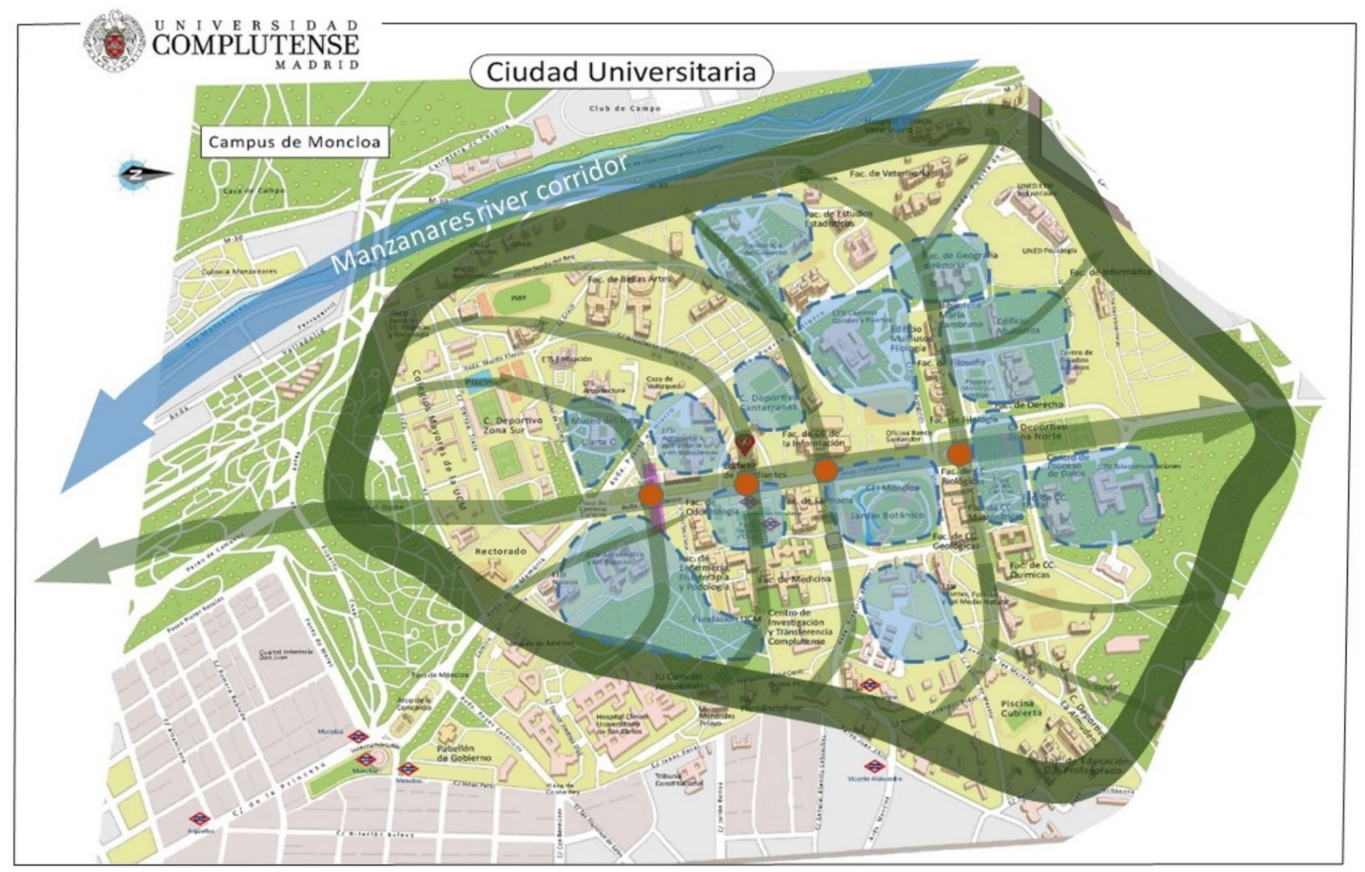
Example of possible main lines of an ecocultural cartography in the UCM main campus. In dark green, the Complutense Green Ring; in blue, environmental stewardship circles; in light green, the ecocultural network (nodes and chains connectors of inter-disciplinary knowledge and green spaces among University Schools). The resulting ecocultural cartography brings a symbolic “apple leaf” shape to the Campus, which may ecologically feed the *Manzanares* (“*Apple Orchards*”) river corridor.

On the promotion of wellbeing, some participatory studies on the mental health and well-being of the university community by the authors of this study have led to the formulation of proposals to enhance synergies of interests and initiatives based on ecocultural approaches (Berry, 2019; Gandarillas et al., 2023; Gandarillas and McCall, 2023). Building a dynamic campus culture through a cohesive territory based on an ecocultural network of spaces for collective cultural, artistic, environmental, scientific, leisure, and sports initiatives represents a powerful strategy for the promotion of students’ and staff’s wellbeing. Ecocultural community management of the territory based on collective innovation and place-building may facilitate the formation of connecting lines and nodes of interdisciplinary integration (see Figure 1 for an example on the UCM campus). This approach offers an ecocultural framework for the campus that may facilitate cognitive and affective mapping, identity, cohesion, and a sense of community within the university. This community development strategy may enhance students’ engagement with the university beyond their academic duties, contributing to the promotion of motivation, care, and well-being regarding their university and their studies.

## 5 Conclusion

The present study points to parenting practices and digital technologies as strong predictors of mental health indicators in the context of learning. Among the parenting factors, parents’ high protection and control appear having a negative impact on mental health within the university students’ learning process. Furthermore, the research reveals a strong association between intensive use of social networks and smartphones with increased levels of anxiety and other mental health problems, having even higher impact than parenting practices.

The influence of parenting practices on mental health have been studied for a considerable period. The study on the impacts of digital technologies is relatively recent, and the results are of significant concern, particularly regarding the influence of certain digital devices and platforms on the rise in mental health problems such as anxiety in young people. The prevalence of mental health issues among university students and the psychosocial factors that contribute to them represent a significant challenge for institutions of higher education. To address this issue, which affects to high proportions of the student population in different countries, institutions must adopt a multifaceted approach that considers the emotional and intellectual needs of students. This entails creating an inclusive environment that fosters mental health through proactive teaching attitudes and activities.

Educational institutions should prioritize community and structural measures that foster the holistic growth of students. This should be achieved by establishing a welcoming environment, providing adequate resources and support, and creating a culture of openness that values well-being and ensures easy access to specialized professional help during times of difficulty. It is crucial that academic institutions recognize the importance of students’ psychological well-being in addition to academic success. By integrating a psychological approach and the dynamics of the university and socio-cultural environment into their priorities, they prevent the stigmatization of mental disorders and promote holistic development. Good mental health among university students enables them to face challenges with resilience, maintain focus, and overcome obstacles on their way to graduation.

The study is not without limitations, some of those are related to the use of a cross-sectional design, which precludes establishing definitive causal relationships. Also, the online administration of the questionnaire implies self-selection of participants, which may introduce sampling bias. The proportional representation in the sample of the study fields, sex and age was tested in order to reduce the biases in the interpretation of the results.

Future research directions include consideration of a longitudinal study to explore how the influences of the factors on mental health maintain over time, and the use of more diverse data collection methods, such as qualitative interviews and objective assessments, to mitigate the biases associated with self-reporting and to provide a more complete and accurate picture of the impact of parenting practices and digital technology use on the mental health of university students.

## Data Availability

The raw data supporting the conclusions of this article will be made available by the authors upon request as appropriate.

## Appendix

**Items of the questionnaire used in the study.**

Difficulties that you think may be hindering your studying and learning:

(1 = nothing or almost nothing; 4 = A lot)

- Bad mood/ Irritability.
- Anxiety/nervous.
- Apathy/discouragement/lack of motivation
- Difficulties in attention and concentration.

Items from the EMBU scale:

(1 = Never or almost never; 4 = A lot of the time)

Parent’s *Care.*

I think that my parents tried to make my adolescence stimulating, interesting, and instructive (for instance by giving me good books, arranging for me to go to camp, taking me to clubs).

When faced with a difficult task, I felt supported by my parents.

Parent’s *Control.*

My parents would punish me strictly, even for trifles (small offenses).

Parent’s *Protection.*

I think that my parents’ anxiety that something might happen to me was unwarranted.

Family economic levels:

1. Low; (2) Low-Middle; (3) Middle; (4) High-Middle; (5) High

Digital technologies in the study. How often do you use the following digital and online tools?

(1 = Never or almost never; 4 = A lot of the time)

- Gamification apps (e.g., Kahoot!)
- Apps of online collaboration with other students
- Online diachronic classes (previously recorded by the professor)
- Social media and networks (e.g., WhatsApp, TikTok, Facebook, Twitter)

Socio-demographic variables included:

Biological sex, age, grade, and field of study.

## References

[ref1] Affrunti N. W. Ginsburg G. S. (2012). Maternal overcontrol and child anxiety: the mediating role of perceived competence. Child Psychiatry Hum. Dev. 43, 102–112. doi: 10.1007/s10578-011-0248-z, PMID: 21874362 PMC3358037

[ref2] Agbaria Q. Mahamid F. (2023). The association between parenting styles, maternal self-efficacy, and social and emotional adjustment among Arab preschool children. Psicologia 36:10. doi: 10.1186/s41155-023-00252-4, PMID: 37099037 PMC10133425

[ref3] Akram W. y Kumar R. (2017). A study on positive and negative effects of social media on society. Int. J. Comput. Sci. Engin.. Vol. 5. doi: 10.26438/ijcse/v5i10.351354

[ref4] Aliberti S. M. Cavallo P. Capunzo M. Brongo S. Giraldi L. Santoro E.. (2019). Relationship between health, lifestyle, psychosocial factors and academic performance: a cross-sectional study at the University of Salerno. Epidemiol. Biostatistics Public Health 16, 1–6. doi: 10.2427/12938

[ref5] Álvarez M. Moral M. V. (2020). Phubbing, uso problemático de teléfonos móviles y de redes sociales en adolescentes y déficits en autocontrol. Health Addict. 20, 113–125. doi: 10.21134/haaj.v20i1.487

[ref6] American College Health Association (2015). American college health association-National College Health Assessment II: Reference group executive summary spring 2015. Hanover, MD: American College Health Association.

[ref7] Arrindell W. A. Akkerman A. Bagés N. Feldman L. Caballo V. E. Oei T. P.. (2005). The short-EMBU in Australia, Spain, and Venezuela. Eur. J. Psychol. Assess. 21, 56–66. doi: 10.1027/1015-5759.21.1.56

[ref8] Arrindell W. A. Perris H. Denia M. Van Der Ende J. Perris C. Kokkevi A.. (1988). The constancy of structure of perceived parental rearing style in Greek and Spanish subjects as compared with the Dutch. Int. J. Psychol. 23, 3–23. doi: 10.1080/00207598808247749

[ref9] Asante K. O. Andoh-Arthur J. (2015). Prevalence and determinants of depressive symptoms among university students in Ghana. J. Affect. Disord. 171, 161–166. doi: 10.1016/j.jad.2014.09.025, PMID: 25305431

[ref10] Batool S. S. (2019). Academic achievement: interplay of positive parenting, self-esteem, and academic procrastination. Aust. J. Psychol. 72, 174–187. doi: 10.1111/ajpy.12280

[ref11] Berry J. W. (2019). Ecocultural psychology. Ecocult. Psychol. 15, 4–16. doi: 10.17759/chp.2019150401

[ref12] Cheng W. Wu C. (2021). Family socioeconomic status and Children’s gender differences in Taiwanese teenagers’ perception of parental rearing behaviors. J. Child Fam. Stud. 30, 1619–1632. doi: 10.1007/s10826-021-01965-9

[ref13] Cortés M. R. Cantón J. Cantón D. (2014). “Desarrollo socioafectivo en el contexto familiar [socioemotional development in the family context]” in Desarrollo socioafectivo y de la personalidad [socioemotional and personality development]. eds. Cortés Arboleda M. R. Cantón Cortés J. C. D. D. (Alianza), 213–257.

[ref14] Del Valle M. Zamora E. V. Khalil Y. Altamirano M. (2020). Rasgos de personalidad y dificultades de regulación emocional en estudiantes universitarios. Psicodebate 20, 56–67. doi: 10.18682/pd.v20i1.1877

[ref15] Dhir A. Yossatorn Y. Kaur P. Chen S. (2018). Online social media fatigue and psychological wellbeing—a study of compulsive use, fear of missing out, fatigue, anxiety and depression. Int. J. Inf. Manag. 40, 141–152. doi: 10.1016/j.ijinfomgt.2018.01.012

[ref16] Díaz-Vicario A. Mercader C. Gairín J. (2019). Uso problemático de las TIC en adolescentes. Revista Electrón. Investig. Educ. 21, 1–11. doi: 10.24320/redie.2019.21.e07.1882

[ref17] Dorrance Hall E. Scharp K. M. Sanders M. Beaty L. (2020). Family communication patterns and the mediating effects of support and resilience on students’ concerns about college. Fam. Relat. 69, 276–291. doi: 10.1111/fare.12386

[ref18] Duke E. Montag C. (2017). Smartphone addiction, daily interruptions and self-reported productivity. Addict. Behav. Rep. 6, 90–95. doi: 10.1016/j.abrep.2017.07.002, PMID: 29450241 PMC5800562

[ref19] El Ansari W. Stock C. Snelgrove S. Hu X. Parke S. Davies S.. (2011). Feeling healthy? A survey of physical and psychological wellbeing of students from seven universities in the UK. Int. J. Environ. Res. Public Health 8, 1308–1323. doi: 10.3390/ijerph8051308, PMID: 21655121 PMC3108111

[ref20] El Madani H. El Harch I. Tachfouti N. El Fakir S. Aalouane R. Berraho M. (2023). Estrés psicológico y sus factores relacionados entre estudiantes de enfermería marroquíes: un estudio transversal. Enferm. Clin. 33, 205–215. doi: 10.1016/j.enfcli.2022.11.004 36822473

[ref21] Elsayed W. (2021). The negative effects of social media on the social identity of adolescents from the perspective of social work. College of Humanities and Science, Ajman university, Ajman, United Arab Emirates. Heliyon, n° 7. 10.1016/j.heliyon.2021.e06327 PMC7905185 33665465

[ref22] Emily G. Lipson S. K. Eisenberg D. (2019). Technology and college student mental health: challenges and opportunities. Front. Psych. 10:246. doi: 10.3389/FPSYT.2019.00246, PMID: 31037061 PMC6476258

[ref23] Erceg T. Flander G. Brezinšćak T. (2018). The relationship between compulsive internet use and symptoms of depression and anxiety in adolescence. Alcoholism and Psychiatric Research 54, 101–112. doi: 10.20471/dec.2018.54.02.02

[ref24] Fass M. E. Tubman J. G. (2002). The influence of parental and peer attachment on college students’ academic achievement. Psychol. Sch. 39, 561–573. doi: 10.1002/pits.10050

[ref25] Forman C. Zeebroeck N. (2019). Digital technology adoption and knowledge flows within firms: can the internet overcome geographic and technological distance? Res. Policy 48:103697. doi: 10.1016/j.respol.2018.10.021

[ref26] Franco N. Pérez M. A. de Dios M. J. (2014). Relación entre los estilos de crianza parental y el desarrollo de ansiedad y conductas disruptivas en niños de 3 a 6 años [relationship between parental parenting styles and the development of anxiety and disruptive behaviors in children aged 3 to 6]. Revista Psicol. Clínica Niños Adolescentes 1, 149–156.

[ref27] Fuentes M. C. Alarcón A. Gracia E. García F. (2015). School adjustment among Spanish adolescents: influence of parental socialization. Cultura Educ. 27, 1–32. doi: 10.1080/11356405.2015.1006847

[ref28] Fuentes M. A. Zelaya D. G. Madsen J. W. (2021). Rethinking the course syllabus: considerations for promoting equality, diversity, and inclusion. Teach. Psychol. 48, 69–79. doi: 10.1177/0098628320959979

[ref29] Gallagher R. (2014). National Survey of College Counseling Centers 2014. International Association of Counseling Services, Alexandria, VA. Available at: http://www.collegecounseling.org/wp-content/uploads/NCCCS2014_v2.pdf.

[ref30] Gandarillas M.A. (1995). Cultura y fisiología: El papel de la crianza infantil en la actividad psicofisiológica: Un estudio multidisciplinar [culture and physiology: The role of child rearing in psychophysiological activity: A multidisciplinary study]. Doctoral dissertation. Universidad Autónoma de Madrid

[ref31] Gandarillas M. A. (2011). Psychosocial correlates of peripheral vegetative activity and coordination. Aletheia, 35–36, 211–230.

[ref32] Gandarillas M. A. (2022). “Un enfoque integral sobre la diversidad de aprendizaje y sus predictores psicosociales en el contexto universitario. Estudio preliminar [an integrated approach to learning diversity and its psychosocial predictors in the university context. Preliminary study]” in Innovación Docente y Prácticas Educativas para una Educación de Calidad [Teaching innovation and educational practices for quality education]. ed. Romero C. (Madrid, Spain: Dykinson), 837–863.

[ref33] Gandarillas M. A. Elvira-Zorzo M. N. Rodríguez-Vera M. (2024). The impact of parenting practices and family economy on psychological wellbeing and learning patterns in higher education students. Psicologia 37:8. doi: 10.1186/s41155-024-00291-5 38446334 PMC10917719

[ref34] Gandarillas M. A. McCall M. K. (2023). Ecocultural networks as grounds for spatial planning. A psychosocial approach applied to coastal development. J. Cult. Heritage Manag. Sustain. Develop. 13, 108–122. doi: 10.1108/JCHMSD-01-2021-0008

[ref35] Gandarillas M. A. Montañes M. (2019). Perfiles psicosociales de usuarios de entornos virtuales: motivaciones, conductas y consecuencias. Universitas psychologica 18, 1–14. doi: 10.11144/Javeriana.upsy18-3.ppue

[ref36] Gandarillas M. A. Elvira-Zorzo M. N. Rodríguez-Vera M. Benito Á. Borgards K. Borja Block R.. (2023). Análisis de la diversidad de aprendizaje y su aprovechamiento en medidas innovadores para la inclusión y el aprendizaje colaborativo [Analysis of learning diversity and its use in innovative measures for inclusion and collaborative learning]. Proyectos de Innovación Docente. Docta Complutense. Available at: https://hdl.handle.net/20.500.14352/87879 (Accessed November 25, 2023).

[ref37] García-Oliva C. Piqueras J. A. Marzo J. C. (2017). Uso problemático de internet, el móvil y los videojuegos en una muestra de adolescentes alicantinos. Salud Drogas 17, 189–200. doi: 10.21134/haaj.v17i2.331

[ref38] Gfellner B. M. Córdoba A. I. (2020). The interface of identity distress and psychological problems in students’ adjustment to university. Scand. J. Psychol. 61, 527–534. doi: 10.1111/sjop.12625, PMID: 32048734

[ref39] Gómez O. Del Rey R. Casas J. A. Ortega R. (2014). Estilos parentales e implicación en bullying [parenting styles and involvement in bullying]. Cult. Educ. 26, 132–158. doi: 10.1080/11356405.2014.908665

[ref40] Gómez O. Del Rey R. Romera E. M. Ortega-Ruiz R. (2015). Los estilos educativos paternos y maternos en la adolescencia y su relación con la resiliencia, el apego y la implicación en acoso escolar [the parenting styles of fathers and mothers in adolescence and their relationship with resilience, attachment, and involvement in school bullying]. Anales Psicol. 31, 979–989. doi: 10.6018/analesps.31.3.180791

[ref41] Gordon M. S. Cui M. (2012). The effect of school-specific parenting processes on academic achievement in adolescence and young adulthood. Fam. Relat. 61, 728–741. doi: 10.1111/j.1741-3729.2012.00733.x

[ref42] Guassi-Moreira J. F. Telzer E. H. (2015). Changes in family cohesion and links to depression during the college transition. J. Adolesc. 43, 72–82. doi: 10.1016/j.adolescence.2015.05.012, PMID: 26058003

[ref43] Guterman O. Neuman A. (2018). Personality, socio-economic status and education: factors that contribute to the degree of structure in homeschooling. Soc. Psychol. Educ. 21, 75–90. doi: 10.1007/s11218-017-9406-x

[ref44] Haidt J. (2024). The anxious generation: How the great rewiring of childhood is causing an epidemic of mental illness. New York: Random House.

[ref45] Hamdi M. A. (2018). University youth dependence on social media for access to information: A survey study at the University of Tabuk, Saudi Arabia (Master's thesis). Middle East University, Department of Journalism and Media, Faculty of Information, Amman, Jordan.

[ref46] Heritage B. Ladeira C. Steele A. R. (2023). The development and pilot of the university student embeddedness (USE) scale for student retention within universities: validation with an Australian student sample. High. Educ. 85, 27–54. doi: 10.1007/s10734-022-00813-z

[ref47] Hernesniemi E. Räty H. Kasanen K. Cheng X. Hong J. Kuittinen M. (2017). Burnout among Finnish and Chinese university students. Scand. J. Psychol. 58, 400–408. doi: 10.1111/sjop.12380, PMID: 28800165

[ref48] Heylighen F. Beigi S. (2023). Anxiety, depression and despair in the information age: the techno-social dilemma. (ECCO Working papers). ECCO VUB.

[ref49] Hodkinson C. (2019). ‘Fear of missing out’ (FOMO) marketing appeals: a conceptual model. J. Mark. Commun. 25, 65–88. doi: 10.1080/13527266.2016.1234504

[ref50] Ibrahim A. K. Kelly S. J. Adams C. E. Glazebrook C. (2013). A systematic review of studies of depression prevalence in university students. J. Psychiatr. Res. 47, 391–400. doi: 10.1016/j.jpsychires.2012.11.015 23260171

[ref51] January J. Madhombiro M. Chipamaunga S. Ray S. Chingono A. Abas M. (2018). Prevalence of depression and anxiety among undergraduate university students in low-and middle-income countries: a systematic review protocol. Syst. Rev. 7:57. doi: 10.1186/s13643-018-0723-8, PMID: 29636088 PMC5894225

[ref52] Jaureguizar J. Bernaras E. Bully P. Garaigordobil M. (2018). Perceived parenting and adolescents’ adjustment. Psicologia 31:8. doi: 10.1186/s41155-018-0088-x, PMID: 32026134 PMC6966945

[ref53] Ji S. Wang H. (2018). A study of the relationship between adverse childhood experiences, life events, and executive function among college students in China. Psicologia 31:28. doi: 10.1186/s41155-018-0107-y, PMID: 32026138 PMC6967049

[ref54] Khalil Y. del Valle M. V. Zamora E. V. Urquijo S. (2020). Dificultades de regulación emocional y bienestar psicológico en estudiantes universitarios [Emotional regulation difficulties and psychological well-being in college students]. Subjetividad Procesos Cognitivos 24, 69–83.

[ref55] Kim Y. Mok S. Y. Seidel T. (2020). Parental influences on immigrant students´ achievement-related motivation and achievement: a meta-analysis. Educ. Res. Rev. 30, 100327–100319. doi: 10.1016/j.edurev.2020.100327

[ref56] Lamis D. A. Ballard E. D. May A. M. Dvorak R. D. (2016). Síntomas depresivos e ideación suicida en estudiantes universitarios: las funciones mediadoras y moderadoras de la desesperanza, los problemas con el alcohol y el apoyo social. J. Clin. Psychol. 72, 919–932. doi: 10.1002/jclp.22295, PMID: 27008096

[ref57] Lardy L. Bressoux P. P. De Clercq M. (2022). Achievement of first-year students at the university: a multilevel analysis of the role of background diversity and student engagement. Eur. J. Psychol. Educ. 37, 949–969. doi: 10.1007/S10212-021-00570-0

[ref58] Lew B. Huen J. Yu P. Yuan L. Wang D. F. Ping F.. (2019). Associations between depression, anxiety, stress, hopelessness, subjective well-being, coping styles and suicide in Chinese university students. PLoS One 14:e0217372. doi: 10.1371/journal.pone.0217372, PMID: 31260454 PMC6602174

[ref59] Lipson S. K. Lattie E. G. Eisenberg D. (2019). Increased Rates of Mental Health Service Utilization by U.S. College Students: 10-Year Population-Level Trends (2007-2017). Psychiatr. Serv. 70, 60–63. doi: 10.1176/appi.ps.201800332 30394183 PMC6408297

[ref60] MacCann C. Jiang Y. Brown L. E. Double K. S. Bucich M. Minbashian A. (2020). Emotional intelligence predicts academic performance: a meta-analysis. Psychol. Bull. 146, 150–186. doi: 10.1037/bul0000219, PMID: 31829667

[ref61] Maccoby E. E. (1992). The role of parents in the socialization of children: a historical review. Dev. Psychol. 28, 1006–1017. doi: 10.1037/0012-1649.28.6.1006

[ref62] Manion K. Shah-Preusser N. Dyck T. Thackeray S. Palahicky S. (2020). Learning in teams: collaboratively guiding the journey. Collected Essays Learn. Teach. 13, 25–40. doi: 10.22329/celt.v13i0.6025

[ref63] Mardia K. V. (1970). Measures of multivariate skewness and kurtosis with applications. Biometrika 57, 519–530. doi: 10.2307/2334770

[ref64] Martineli A. K. B. Pizeta F. A. Loureiro S. R. (2018). Behavioral problems of school children: impact of social vulnerability, chronic adversity, and maternal depression. Psicologia 31:11. doi: 10.1186/s41155-018-0089-9, PMID: 32026068 PMC6967022

[ref65] Matalinares M. L. Díaz G. Ornella R. Deyvi B. Uceda J. Yaringaño J. (2016). Afrontamiento del estrés y bienestar psicológico en estudiantes universitarios de Lima y Huancayo. Persona 19, 105–106.

[ref66] Mathieu S. L. Conlon E. G. Waters A. M. Farrell L. J. (2020). Perceived parental rearing in Paediatric obsessive–compulsive disorder: examining the factor structure of the EMBU child and parent versions and associations with OCD symptoms. Child Psychiatry Hum. Dev. 51, 956–968. doi: 10.1007/s10578-020-00979-6 32146572

[ref67] Mi Z. Cao W. Diao W. Wu M. Fang X. (2023). The relationship between parental phubbing and mobile phone addiction in junior high school students: a moderated mediation model. Front. Psychol. 14:1117221. doi: 10.3389/fpsyg.2023.1117221, PMID: 37123292 PMC10132137

[ref68] Mirza A. A. Baig M. Beyari G. M. Halawani M. A. Mirza A. A. (2021). Depression and anxiety among medical students: a brief overview. Adv. Med. Educ. Pract. 12, 393–398. doi: 10.2147/AMEP.S302897, PMID: 33911913 PMC8071692

[ref69] Molina M. Raimundi M. Bugallo L. (2017). La percepción de los estilos de crianza y se relación con las autopercepciones de los niños de Buenos Aires: Diferencias en función del género [The perception of parenting styles and its relationship with self-perceptions in children from Buenos Aires: Differences based on gender]. Universitas Psychol. 16, 1–12. doi: 10.11144/Javeriana.upsy16-1.pecr

[ref70] Moral J. E. Urchaga J. D. Ramos A. J. Maneiro R. (2020). Relationship of parental support on healthy habits, school motivations and academic performance in adolescents. Int. J. Environ. Res. Public Health 17, 1–15. doi: 10.3390/ijerph17030882, PMID: 32023826 PMC7037333

[ref71] Morales F. M. Pérez J. M. (2019). The role of anxiety, coping strategies, and emotional intelligence on general perceived self-efficacy in university students. Front. Psychol. 10:1689. doi: 10.3389/fpsyg.2019.01689, PMID: 31447720 PMC6692438

[ref72] Moreira de Freitas R. Carvalho Oliveira T. Lopes de Melo J. do Vale e Silva J. de Oliveira e Melo K. Fontes Fernandes S. (2021). Percepciones de los adolescentes sobre el uso de las redes sociales y su influencia en la salud mental. Enfermería global 20, 324–364. doi: 10.6018/eglobal.462631

[ref73] Muñoz-Miralles R. Ortega-González R. Batalla-Martínez C. López-Morón M. R. Manresa J. M. Torán-Monserrat P. (2014). Acceso y uso de nuevas tecnologías entre los jóvenes de educación secundaria, implicaciones en salud. Revista Atención Primaria 46, 77–88. doi: 10.1016/j.aprim.2013.06.001, PMID: 24035765 PMC6983583

[ref74] Nagib N. Horita R. Miwa T. Adachi M. Tajirika S. Imamura N.. (2023). Impact of COVID-19 on the mental health of Japanese university students (years II-IV). Psychiatry Res. 325:115244. doi: 10.1016/j.psychres.2023.115244, PMID: 37182282

[ref75] Nahar Z. Eqbal S. Supti K. F. Hasan A. H. M. N. Kawsar A. B. M. R. Islam M. R. (2023). A dataset on the prevalence and associated risk factors for mental health problems among female university students in Bangladesh. Data Brief 48:109203. doi: 10.1016/j.dib.2023.109203 37213555 PMC10197013

[ref76] Njega M. W. Njoka J. N. Ndung C. W. (2019). Relationship between psychosocial dynamics and academic performance of secondary school students: a comparative study between Murang’a and Kirinyaga counties, Kenya. J. Arts Human. 8, 42–52.

[ref77] Olorunsogo T. O. Balogun O. D. Ayo-Farai O. Ogundairo O. Maduka C. P. Okongwu C. C.. (2024). Mental health and social media in the US: a review: investigating the potential links between online platforms and mental well-being among different age groups. World J. Advanced Res. Rev. 21, 321–334. doi: 10.30574/wjarr.2024.21.1.0015

[ref78] Orzech K. Grandner M. Roane B. Carskadon M. (2016). Digital media use in the 2 h before bedtime is associated with sleep variables in university students. Comput. Hum. Behav. 55, 43–50. doi: 10.1016/j.chb.2015.08.049, PMID: 28163362 PMC5279707

[ref79] Piccolo L. D. R. Arteche A. X. Fonseca R. P. Grassi-Oliveira R. Salles J. F. (2016). Influence of family socioeconomic status on IQ, language, memory and executive functions of Brazilian children. Psicologia 29:23. doi: 10.1186/s41155-016-0016-x

[ref80] Poorolajal J. Ghaleiha A. Darvishi N. Daryaei S. Panahi S. (2017). E prevalence of psychiatric distress and associated risk factors among college students using GHQ-28 questionnaire. Iran. J. Public Health 46, 957–963. 28845407 PMC5563878

[ref81] Regalado Chamorro M. Medina Gamero A. Tello Cabello R. (2022). La salud mental en adolescentes: internet, redes sociales y psicopatología [mental health in adolescents: internet, social networks and psychopathology]. Atención primaria 54:102487. doi: 10.1016/j.aprim.2022.102487, PMID: 36257099 PMC9583028

[ref82] Robledo P. García J. N. (2009). El entorno familiar y su influencia en el rendimiento académico de los alumnos con dificultades de aprendizaje: revisión de estudios empíricos [the family environment and its influence on the academic performance of students with learning difficulties: a review of empirical studies]. Aula Abierta 37, 117–128.

[ref83] Rock A. Barrington C. Abdoulayi S. Tsoka M. Mvuls P. Handa S. (2016). Social networks, social participation, and health among youth living in extreme poverty in rural Malawi. Soc. Sci. Med. 170, 55–62. doi: 10.1016/j.socscimed.2016.10.005, PMID: 27760393 PMC5350075

[ref84] Rodríguez-Hernández C. Cascallar E. Kyndt E. (2020). Socio-economic status and academic performance in higher education: a systematic review. Educ. Res. Rev. 29, 100305–100324. doi: 10.1016/j.edurev.2019.100305

[ref85] Rodríguez-Hidalgo A. J. Pincay A. A. Payán A. M. Herrera-López M. Ortega-Ruiz R. (2021). Los Predictores Psicosociales del Bullying Discriminatorio Debido al Estigma Ligado a las Necesidades Educativas Especiales (NEE) y la Discapacidad. Psicol. Educ. 27, 187–197. doi: 10.5093/psed2020a22

[ref86] Rojo-Ramos J. Manzano-Redondo F. Barrios-Fernandez S. Garcia-Gordillo M. A. Adsuar J. C. (2021). A descriptive study of specialist and non-specialist teachers’ preparation towards educational inclusion. Int. J. Environ. Res. Public Health 18:7428. doi: 10.3390/ijerph18147428 34299880 PMC8304054

[ref87] Sales S. S. da Costa T. M. Gai M. J. P. (2021). Adolescentes en la era digital: impactos en la salud mental. Investig. Soc. Desarrollo 10:e15110917800. doi: 10.33448/rsd-v10i9.17800

[ref88] Samaniego P. A. Buenahora B. A. (2016). Variables relacionadas con ansiedad social en adolescentes: un modelo de regresión lineal múltiple. Interacciones 2, 109–122. doi: 10.24016/2016.v2n2.40

[ref89] Santander J. Berner J. E. Contreras A. M. Gómez T. (2013). Prevalencia de déficit atencional en estudiantes de medicina de la Pontificia Universidad Católica de Chile. Rev. Chil. Neuropsiquiatr. 51, 169–174. doi: 10.4067/S0717-92272013000300002

[ref90] Suárez Monzón N. Requeiro Almeida R. Heredia Gálvez S. A. Lara Paredes D. G. (2022). Salud mental y usos de la tecnología en el contexto universitario. Una Revisión Literatura 52, 191–228. doi: 10.30827/publicaciones.v52i3.22272

[ref91] Tan G. X. Soh X. C. Hartanto A. Goh A. Y. Majeed N. M. (2023). Prevalence of anxiety in college and university students: an umbrella review. J. Affect. Disord. Reports 14:100658. doi: 10.1016/j.jadr.2023.100658

[ref92] Tian-Ci Quek T. Wai-San Tam W. X. Tran B. Zhang M. Zhang Z. Su-Hui Ho C.. (2019). The global prevalence of anxiety among medical students: a meta-analysis. Int. J. Environ. Res. Public Health 16:2735. doi: 10.3390/ijerph16152735, PMID: 31370266 PMC6696211

[ref93] Tinajero C. Martínez-López Z. Rodriguez M. Páramo M. (2020). Perceived social support as a predictor of academic success in Spain university students. Anales Psicología 36, 134–142. doi: 10.6018/analesps.344141

[ref94] Törnberg P. Andersson C. Lindgren K. Banisch S. (2021). Modeling the emergence of affective polarization in the social media society. PLoS One 16:e0258259. doi: 10.1371/journal.pone.0258259, PMID: 34634056 PMC8504759

[ref95] Tran A. Tran L. Geghre N. Darmon D. Rampal M. Brandone D.. (2017). Health assessment of French university students and risk factors associated with mental health disorders. PLoS One 12:e0188187. doi: 10.1371/journal.pone.0188187, PMID: 29176864 PMC5703533

[ref96] Trunce S. T. Villarroel G. D. P. Arntz J. A. Muñoz S. I. Werner K. M. (2020). Niveles de depresión, ansiedad, estrés y su relación con el rendimiento académico en estudiantes universitarios. Investig. Educ. Médica 9, 8–16. doi: 10.22201/fm.20075057e.2020.36.20229

[ref97] Vaterlaus J. M. Patten E. V. Roche C. Young J. A. (2015). Gettinghealthy: the perceived influence of social media on young adult health behaviors. Comput. Hum. Behav. 45, 151–157. doi: 10.1016/j.chb.2014.12.013

[ref98] Vera-Villarroel P. Celis-Atenas K. Urzúa A. S. Contreras D. Lillo S. (2016). Los afectos como mediadores de la relación optimismo y bienestar. Revista Argentina Clínica Psicol. 25, 195–202.

[ref99] Walsh B. A. Mitchell S. Batz R. Lee A. Aguirre M. Lucero J.. (2023). Familial roles and support of doctoral students. Fam. Relat. 72, 2444–2464. doi: 10.1111/fare.12848

[ref100] Walther J. B. (2022). Social media and online hate. Curr. Opin. Psychol. 45:101298. doi: 10.1016/j.copsyc.2021.12.010 35158213

[ref101] Wang H. Y. Leif C. C. (2018). Digital nativity and information technology addiction: age cohort versus individual difference approaches. Comput. Hum. Behav. 90, 1–9. doi: 10.1016/j.chb.2018.08.031

[ref102] Wolfers L. Festl R. Utz S. (2020). Do smartphones and social network sites become more important when experiencing stress? Results from longitudinal data. Comput. Hum. Behav. 109:106339. doi: 10.1016/j.chb.2020.106339, PMID: 32747849 PMC7224514

[ref103] World Health Organization (2001). Fortaleciendo la prevención de salud mental. Ginebra: World Organization Health.

[ref104] World Health Organization (2014). Invertir en Salud mental. Ginebra: World Organization Health.

[ref105] Yangzong C. Lerkiatbundit S. Luobu O. Cui C. Liabsuetrakul T. Kangzhuo B.. (2016). Validity and reliability of the Tibetan version of s-EMBU for measuring parenting styles. Psychol. Res. Behav. Manag. 10, 1–8. doi: 10.2147/PRBM.S111073, PMID: 28053560 PMC5189697

[ref106] Yongmei H. Jiaying L. (2022). Psychometric evaluation of EMBU for junior high school Students in Guangdong Province. Int. J. Arts Soc. Sci. 5, 13–19.

